# Advanced and Conventional Magnetic Resonance Imaging in Neuropsychiatric Lupus

**DOI:** 10.12688/f1000research.6522.2

**Published:** 2015-07-28

**Authors:** Nicolae Sarbu, Núria Bargalló, Ricard Cervera

**Affiliations:** 1 Section of Neuroradiology, Department of Radiology, Hospital Clinic, Barcelona, Catalonia, 08036, Spain; 2 Magnetic Resonance Imaging Core Facility, Institut d'Investigacions Biomèdiques August Pi i Sunyer (IDIBAPS), Barcelona, Catalonia, 08036, Spain; 3 Department of Autoimmune Diseases, Hospital Clinic, Barcelona, Catalonia, 08036, Spain

**Keywords:** neuropsychiatric lupus, NPSLE, systemic lupus erythematosus, SLE, magnetic resonance imaging, MRI

## Abstract

Neuropsychiatric lupus is a major diagnostic challenge, and a main cause of morbidity and mortality in patients with systemic lupus erythematosus (SLE). Magnetic resonance imaging (MRI) is, by far, the main tool for assessing the brain in this disease. Conventional and advanced MRI techniques are used to help establishing the diagnosis, to rule out alternative diagnoses, and recently, to monitor the evolution of the disease. This review explores the neuroimaging findings in SLE, including the recent advances in new MRI methods.

## Introduction

Despite the fact that the outcome of patients with systemic lupus erythematosus (SLE) has improved considerably over the last decades, neuropshychiatric involvement remains a main cause of morbi-mortality ^1, 2^, being responsible for up to 19% of deaths in SLE ^3, 4^. The real prevalence of neuropsychiatric SLE (NPSLE) remains unknown, with significant heterogeneity between studies, from 14% to 95% depending on the inclusion criteria; an average of 40–50% is probably widely accepted ^5– 8^. Reliable methods for diagnosing NPSLE are also unknown, the clinical judgment remaining the cornerstone for differentiation of these patients ^9, 10^. Therefore, NPSLE represents a major diagnostic challenge, being essentially a diagnosis of presumption and exclusion, established after having ruled out other possible causes such as trauma, infection, drug effects, epilepsy, migraine, psychiatric disorders, multiple sclerosis, posterior reversible encephalopathy and previous nervous system disorders ^5, 6, 11, 12^. Very recently, an algorithm based on a probability score was validated to determine the relationship between neuropsychiatric involvement and SLE ^13^. On the other hand, reaching the correct diagnosis of NPSLE is critical in terms of therapeutic decisions and outcome.

According to 1999 American College of Rheumatology (ACR) Case Definitions for NPSLE, 19 neuropsychiatric syndromes are defined, divided into 12 central and 7 peripheral ^14^. The central ones are further divided into neurological (aseptic meningitis, cerebrovascular disease, demyelinating syndrome, headache including migraine and benign intracranial hypertension, movement disorders, myelopathy, epilepsy), and psychiatric (acute confusional states, anxiety disorder, cognitive dysfunction, affective disorder). The peripheral syndromes are acute inflammatory demyelinating polyradiculopathy (Guillain-Barre syndrome), autonomic disorder, mononeuropathy (single/multiplex), myasthenia gravis, cranial neuropathy, plexopathy, and polyneuropathy. The most common syndromes which require neuroimaging studies are headache, cerebrovascular disease, epilepsy and cognitive dysfunction ^8, 15^, and also represent four out of five globally most prevalent NPSLE syndromes, as demonstrated by an extensive, recent meta-analysis ^7^. NPSLE is also divided into primary, attributed to SLE specific mechanisms, and secondary, consequence of infections, drugs or metabolic errors, although there are no definitive methods to differentiate between them ^6, 10^.

In spite of outstanding advances and increasing efforts into research, the physiopathology of NPSLE remains still unclear. Neural and vascular injuries related to antibodies and cytokines were incriminated in active NPSLE. The pathological substrate of NPSLE consists of microangiopathic disease (the most frequent neuropathological finding, typically multifocal, due to intimal hyperplasia, erythrocytes extravasation and fibrin thrombi), macroscopic infarcts (partially explained by secondary coagulopathy due to antiphospholipid antibodies or by embolic phenomena due to Libman–Sacks endocarditis), accelerated atherosclerosis (partially due to steroid treatment, vasculitis and microhemorrhages), direct immune mediated alterations, demyelination and microembolisms ^5, 16– 19^.

Magnetic resonance imaging (MRI) is the gold standard for studying the brain in SLE. The role of other imaging modalities such as computer tomography (CT) is essentially to rule out acute complications such as hemorrhage or large infarcts, or to assess differential diagnoses ^5, 20, 21^. The large spectrum of clinical presentations, laboratory and pathological findings in NPSLE made the neuroradiological findings nonspecific, a wide range of abnormalities being described ^8, 22^. The most frequently reported findings with conventional MRI in large series of NPSLE were multiple small white-matter lesions (30–75%) and cortical atrophy (15–20%), although there is a large percentage of patients (25–60%) with normal MRI scan ^8, 11, 23, 24^. Advanced MRI techniques such diffusion-tensor, magnetization-transfer and volumetric studies, which give microstructural and functional information, could provide evidence of subtle brain changes that allow better understanding of the NPSLE mechanisms. Furthermore, the correlation of the neuroradiological, clinical and immunological biomarkers could give insights into the pathophysiology of the disease. The present review aims to describe the neuroimaging findings in conventional and advanced MRI imaging in NPSLE patients, and their importance from a practical point of view.

### Conventional MRI neuroimaging findings

Around 50% of the NPSLE patients had normal MRI, especially in diffuse syndromes such as headache, mood disorder, and psychiatric disease ^8^. In the other half of the patients, the most common neuroimaging findings can be classified as vascular diseases (small or large vessel disease), and inflammatory-type lesions ( Table 1).

**Table 1.** Magnetic resonance imaging classification proposed for brain abnormalities in patients with neuropsychiatric lupus.

Abnormalities type
Inflammatory-like lesions Location: supratentorial/infratentorial Contrast-enhancement Diffusion restriction Large vessel disease Single/multiple Acute/subacute/chronic Vascular territory Small vessel disease ^28^ White matter hyperintensities ^29^ Location: frontal, parieto-occipital, temporal, basal ganglia, infratentorial Degree of involvement: focal lesions, beginning confluence, diffuse involvement Lesion burden: <5 lesions (low lesion burden); 5–25 (intermediate lesion burden); >25 (high lesion burden) Lacunes Recent small subcortical infarcts Microbleeds Brain atrophy (GCA scale) ^49^

Abbreviations: GCA-Global Cortical Atrophy scale.

**Figure 1a–b.**
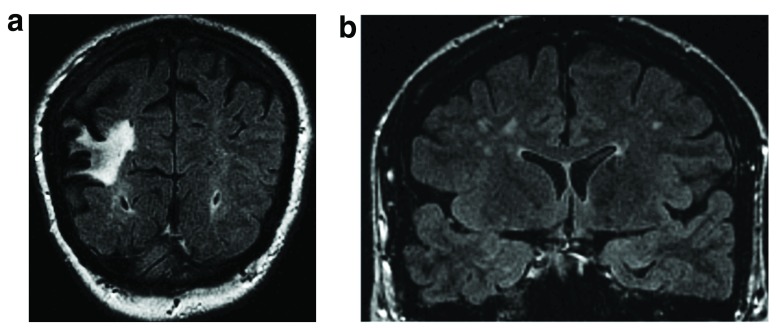
Coronal FLAIR images demonstrate both large and small vessel disease. **1a** Large hyperintense cortico-subcortical area consistent with a chronic stroke involving the right middle cerebral artery territory. **1b** Focal bilateral white matter hyperintensities reflecting small vessel disease. Figure origin: Department of Radiology, Hospital Clinic Barcelona.

Vascular disease, although nonspecific and in many forms of manifestation, is the hallmark of NPSLE ^8^. Vascular lesions are ill-defined hyperintensities on T2, and moderately hypointense or isointense on T1. Large vessel disease refers to large infarcts, which have medium-to-large size, are roughly wedge-shaped, occur with a vascular territory distribution, and involve both grey and white matter ( Figure 1a). With diffusion-weighted imaging (DWI) it is possible to determine if they are in the acute, subacute or chronic stage, including silent infarcts. Large vessel infarcts are one of the most debilitating complications of NPSLE, and are found in 10–15% of patients and at a mean age of 35–40 years ^8, 23, 25, 26^. When infarcts occur in NPSLE, a tendency to multiplicity was noticed, which is translated into a high recurrence of ischemic events ^8^. Middle cerebral artery territory is mainly involved, as in the general population ^8^. Many authors associated antiphospholipid antibodies with infarcts and reported a stroke recurrence of around 50% when these antibodies were present ^23, 25, 27^. Stroke was also more commonly observed in the presence of hypertension, cerebrovascular syndrome and seizures ^5^.

Small vessel disease is typically represented by lesions smaller than 1 cm, which follow the distribution of the white matter (periventricular, deep, subcortical) ( Figure 1b). Recently, the definitions of neuroimaging findings of small vessel disease have been established and consist in white-matter hyperintensities, recent small subcortical infarcts, lacunes, microbleeds and brain atrophy ^28^. White-matter hyperintensities (WMH) are the most widespread type of small vessel disease seen in SLE patients, and represents the collective term referring to small T2-hyperintensities including the white matter, basal ganglia, cerebellum and brainstem ^28^. They are characterized as hyperintense on T2 and FLAIR sequences, without cavitation, generally small and ill-defined ^28, 29^. The differential diagnosis of WMH is very wide, being associated with many conditions including ageing, dyslipidemia, diabetes, hypertension, heart diseases and migraine ^28^. However, many previous reports already proved increased frequency of WMH in SLE and NPSLE ^22, 30– 37^. WMH had been shown to involve preferentially the frontal and parietal lobes, consistent with an anterior to posterior gradient, similar to other causes of WMH, but different from inflammatory demyelinating etiologies such as multiple sclerosis ^8, 38^. In a quantitative cerebral MRI assessment, Appenzeller *et al.* ^35^ showed that age, duration of neuropsychiatric manifestations and cumulative corticosteroid dosage were independent predictors for WMH in SLE. In a recent study in patients with newly diagnosed SLE, WMH were found in 8% of the patients ^39^. Nevertheless, these lesions were observed more frequently in NPSLE when compared with SLE without neuropsychiatric manifestations, with average ranges from 40 to 60% ^8, 11, 20, 35, 37, 39, 40^. WMH were associated with cerebrovascular disease, cognitive dysfunction, seizures, antiphospholipid antibodies, low complements (C3, C4, CH50), age, disease duration, and cumulative corticosteroid dose ^8, 35^. Previous reports demonstrated a significant association between both NPSLE activity (Neuro-SLEDAI) and injury (Neuro-SLICC) scores with the number of WMH (high lesion burden) ^11, 35, 37, 39, 41^. Furthermore, new lesions were noticed during onset of new neuropsychiatric manifestations, and resolution of lesions was found with clinical improvement ^26, 42, 43^. Quantitative methods are increasingly proposed for the quantification and follow-up of the WMH in NPSLE, as they can function as an independent predictor for the NPSLE activity and injury, holding promise to open a new line of follow-up of NPSLE patients and their response to therapy, similar to the monitoring of multiple sclerosis ^33, 35, 39^.

Recent small subcortical infarcts, commonly known as lacunar infarcts, are infarctions in the territory of perforating arterioles, of less than 20 mm in its maximum diameter in the axial plane, with imaging signs or clinical symptoms consistent with a lesion occurring in the previous few weeks ^28^. Their natural evolution is into lacunes, WMH without cavitation, or they might disappear ^44^. Old lacunar infarcts (lacunes) must be differentiated from perivascular (Virchow-Robin) spaces, which generally are smaller, located mostly around the anterior commissure and usually appear linear when imaged parallel to the course of the vessel. Lacunes were commonly described in elderly, asymptomatic individuals, in the presence of hypertension, and were related to dementia, gait impairment and increased risk of stroke ^28^. Very few studies evaluated lacunes in NPSLE and they were found with a prevalence of 11.5–16%, higher than in the general population ^8, 11^. Cerebral microbleeds are small (usually 2–5 mm, but up to 10 mm) round or oval areas of signal void with associated blooming on paramagnetic-sensitive sequences such as T2*-weighted gradient recalled echo (GRE) or susceptibility-weighted images (SWI). Microscopically, hemosiderin-laden macrophages in perivascular tissue are seen, indicating vascular leakage of blood cells, related to bleeding-prone microangiopathy. In the general population, microbleeds are usually located in the cortico-subcortical junction, deep grey and white matter, brainstem and cerebellum. They were associated with hypertension, amyloid angiopathy, cognitive impairment and Alzheimer disease ^45, 46^. In NPSLE, microbleeds were found in 14.5% of the patients on GRE/SWI sequences, and were correlated with lupus anticoagulant (antiphospholipid antibodies) and cerebrovascular syndrome ^8^.

Cortical atrophy is seen as generalized enlargement of peripheral cerebrospinal fluid spaces and is best evaluated on volumetric 3D-T1 or FLAIR images. In the general population, age related atrophic changes are small prior to age 50 years, as proved by a large study ^47^ or, similarly, by another underlying that brain volumes in females remained stable over a span of 15 to 69 years of age ^48^. There are different scales to unify the radiological language, one of the most known being the global cortical atrophy scale (GCA) ^49^. GCA evaluation at the onset of NPSLE observed cortical atrophy in 18.5% of the subjects, most commonly in a mild grade, and at a mean age of 42.5 years ^8^. Brain atrophy occurs more frequently in the presence of other radiological manifestations consistent with small vessel disease, such as WMH, high lesion burden, lacunes and microbleeds ^8^. Brain atrophy was also correlated with lupus anticoagulant, low complement, longer disease duration, cognitive dysfunction and cerebrovascular disease ^23, 37^. Many authors suggested that the atrophy might be the result of the prednisone use ^37, 50^, while others found no association ^31, 39, 51, 52^, which suggests that additional mechanisms, probably related to NPSLE itself, seem to be involved ^53– 56^.

Less frequently, some NPSLE patients present inflammatory-type lesions which were described as ill defined, hyperintense on T2 and FLAIR, involving the grey and white matter, generally medium or large-sized, some of them with contrast enhancement or diffusion restriction, without vascular territory distribution nor clinical and radiological features of infarcts, which usually resolves after aggressive corticosteroid treatment. They were reported in 5–10% of patients, and were correlated with low complement levels, indicating an immunological damage related to antibodies and cytokines and supporting the immunological pathogenesis of NPSLE ^6^. Yet rarely present, findings related to cerebral vasculitis were described, when angiography exams (MRI or conventional) could show focal beadings and narrowings of large and small arteries ^16, 57– 60^.

**Figure 2.**
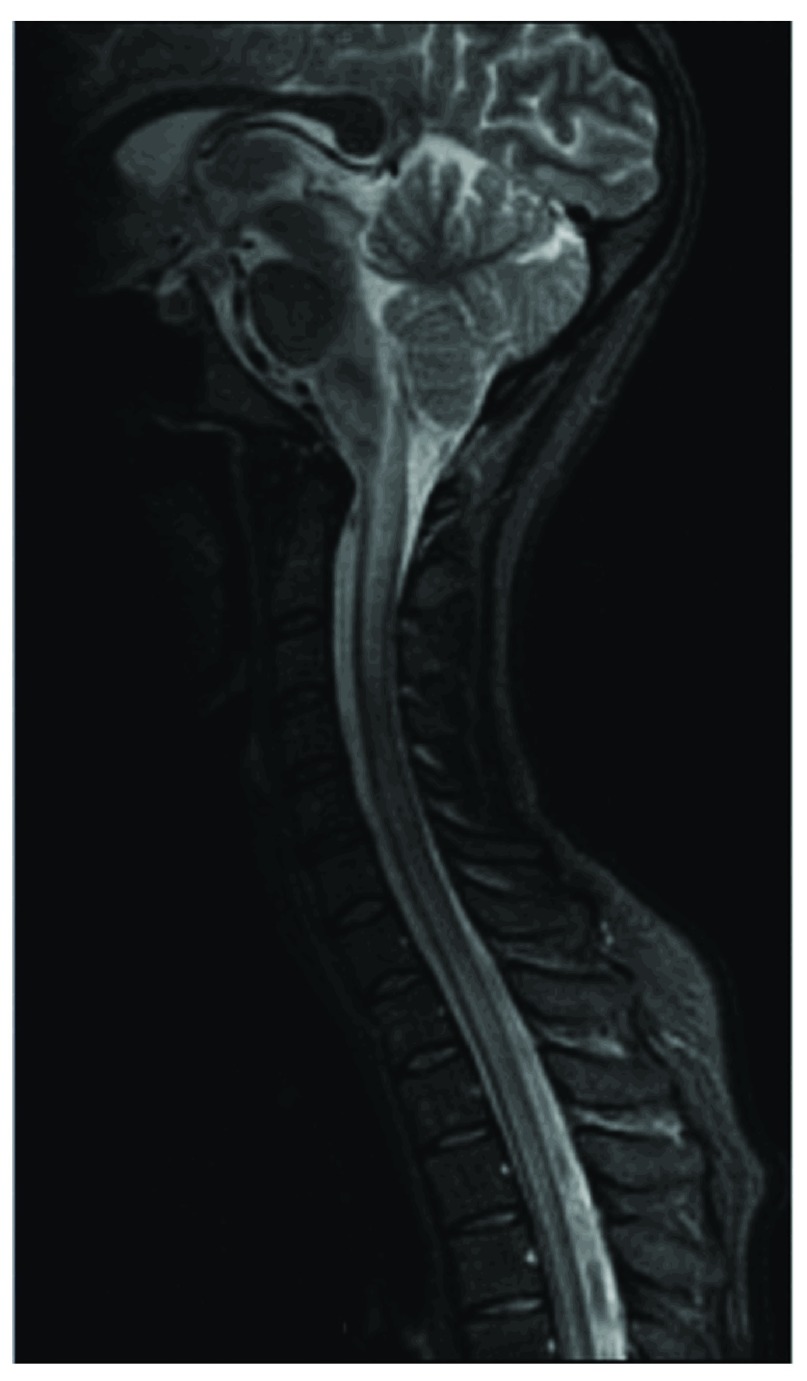
Sagittal T2-weighted image shows an extensive hyperintensity involving the medulla oblongata and the cervico-toracic cord, compatible with a myelitis pattern. Figure origin: Department of Radiology, Hospital Clinic Barcelona.

Myelitis, a type of inflammatory involvement of the central nervous system, is one of the most debilitating complications of NPSLE and occurs in 1–5% of SLE patients. It usually develops early in the evolution of the disease and associates a worse outcome. In 39% of the patients with SLE related myelopathy, it constitutes the presenting symptom of SLE, and in another 42% it occurs during the first 5 years after the diagnosis. The most described MRI pattern in SLE myelitis is consistent with transverse myelitis: commonly long affected segment, more than 2–3 vertebral bodies in length and with injury of both halves of the cord ( Figure 2). Transverse myelitis associates a variable swelling and focal enlargement. Enhancement is usually absent or poor, patchy in the most active presentations. The outcome of SLE myelitis is variable, ranging from complete recovery to severe disability, but the injury is typically much less extensive on follow up MRI ^10, 61^.

### Advanced MRI techniques

Up to 40–50% of NPSLE patients had no brain abnormalities on conventional MRI ^8, 11, 31, 33, 36, 62^. Nonetheless, advanced MRI sequences in NPSLE demonstrated underlying abnormalities in normal appearing white and grey matter, which shows the limitations of conventional sequences ^52, 63, 64^. Recent studies used advanced MRI techniques in the analysis of NPSLE, as the assessment of tissue-specific atrophy by morphometric methods ^38, 52, 65^, diffusion-tensor imaging ^52, 64, 66, 67^, magnetization transfer imaging ^52, 66, 67^, magnetic resonance spectroscopy ^67, 68^ and perfusion MRI. The use of nanoparticles for molecular MRI is another new technique with potential interest in SLE, although its applications in NPSLE have not yet been studied ^69^.

Voxel based morphometry (VBM) is a technique which allows the assessment of the focal differences in brain anatomy and, therefore, the assessment of tissue-specific atrophy. The volume in every voxel is compared across the brain, and VBM is frequently performed for examining differences between populations, although it can also be used to assess asymmetries between brain hemispheres. Morphometric studies showed that decreased whole brain volume with increased lateral ventricle volume and both global gray matter and white matter atrophy are present in SLE patients compared to healthy controls ^70^. Moreover, it was demonstrated that atrophy evolved over a short period of time ^52, 68, 71^. A number of publications found that selective cortical atrophy was the tissue specific atrophy measure with best correlation with the presence of NPSLE, and suggested that cortical atrophy is more important for mediating brain damage in NPSLE patients than the white matter lesions ^52, 71^. From a practical point of view, the macroscopic damage of the cortical gray matter might be more important for identifying NPSLE patients than the micro- or macrostructural damage in the white matter ^52, 71^, yet it was reported an association of NPSLE with both cortical and central atrophy ^52, 65, 72^. Some authors compared cohorts of NPSLE with SLE and controls. The NPSLE group exhibited decreased cortical thickness in left frontal and parietal lobes as well as in right parietal and occipital lobes compared to controls. Both SLE and NPSLE groups exhibited comparable thinning in frontal and temporal lobes ^73^. Automated morphometric methods were also used for segmenting white matter lesions in patients with SLE, which could give a more precise quantification of the focal injuries ^74^.

**Figure 3a–b.**
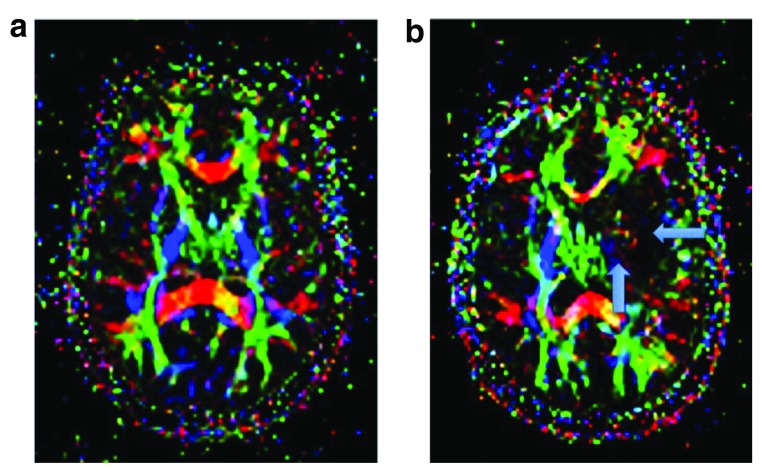
Axial maps of fractional anisotropy (FA). **3a** Normal FA shows the integrity and directionality of the white matter fibers (red: right-left, green: anterior-posterior, blue: craneo-caudal). **3b** Altered (low) FA seen as loss of the normal colors of the left corticospinal tract in the internal capsule and of the left longitudinal fasciculus related to ischemic infarct of the territory of the left middle cerebral artery *(arrows)* in a patient with neuropsychiatric lupus and stroke. Figure origin: Department of Radiology, Hospital Clinic Barcelona.

Diffusion-tensor imaging (DTI) is based on the measurement of water diffusion through cellular compartments, and was demonstrated to provide better resolution than conventional sequences regarding white matter microstructure ( Figure 3a–b) ^75, 76^. Compared to more isotropic movement of water in gray matter, the diffusion in white matter presents higher anisotropy, with preferential diffusion along the length of the axon. This anisotropy is due to the well-structured axonal membranes and their myelin sheaths. The diffusion can be quantified by the following parameters: apparent diffusion coefficient (ADC), fractional anisotropy (FA), mean diffusivity (MD), radial diffusivity (RD) and axial diffusivity (AD). FA is a measure of myelination and axonal integrity, and MD a measure of molecular motion. High FA and low MD suggest greater myelination and axonal integrity. Previous studies found changes in various DTI indices in SLE and NPSLE patients, in relation to important microscopic injury of the white matter ^77^. In patients with SLE, white matter injury in frontal lobes, corpus callosum, and thalamus has been found ^68, 78– 80^. FA values were reported to be lower and MD values to be higher in the brain of NPSLE patients than in healthy controls. Increased AD of white matter was also correlated with NPSLE when compared to healthy populations. It was suggested that the underlying pathological substrate of white matter changes in NPSLE may be the selective axonal damage ^52, 66, 71^. A localized injury of white matter tracts was also demonstrated in the limbic system, internal capsule, corpus callosum, forceps major and corona radiata ^64, 67, 79, 81, 82^. Very recent publications underline the role of DTI as an imaging biomarker of NPSLE ^83^.

Magnetization transfer imaging (MTI) is based on the interaction between free water protons and bound protons. The differences in the proton mobility in various macromolecules and tissues are used to generate differences in image signal. Thus, MTI is used to generate contrast, and it has a variety of clinical applications. Volumetric MTI was used to quantify cerebral lesions in different diseases, mainly in multiple sclerosis. Bosma *et al.* ^84^ compared MTI histogram parameters in 5 groups of patients: active NPSLE, chronic NPSLE, SLE without NPSLE, multiple sclerosis, and normal control subjects. The magnetization transfer ratio histograms in the group of SLE without NPSLE and the group of healthy controls were similar, whereas those in chronic NPSLE and multiple sclerosis groups were flatter. The active NPSLE group showed also a flattening of the histograms, but with a higher magnetization transfer ratio. This suggests that MTI could be able to differentiate active NPSLE. It is also believed that MTI might be a good method for monitoring treatment trials in NPSLE ^84^. A report combining MTI with magnetic resonance spectroscopy (MRS) found correlation between brain atrophy and MRS markers of axonal and myelin damage ^67^. Studies combining MTI with DWI, MRS and T2 relaxometry data in NPSLE suggest a common pathogenesis in NPSLE in spite of the many differences in the neuropsychiatric presentation ^52, 66^.

MRS allows the analysis of brain metabolites. Different proton groups have different magnetic fields in relation to their valence electrons. As a result, they resonate at different frequencies of the magnetic field, which can be demonstrated by MRS, as peaks that correspond to different metabolites. N-acetylaspartate (NAA) is one of the main markers assessed on MRS and is found in higher concentrations in neurons, thus it is a marker of neuronal viability. Glutamate, a non-essential amino acid, is the most important excitatory neurotransmitter, and prolonged neuron excitation by glutamate can be toxic to neurons. NAA and glutamine-glutamate changes were demonstrated in normal-appearing brain in SLE patients, before neurologic and imaging manifestations became apparent, which suggests that these markers might predict the early cerebral involvement of SLE ^85^. Lower NAA ratios were also reported in both SLE and NPSLE patients ^63^, and increased myo-inositol, a marker of gliosis, was suggested as a marker of poor prognosis in NPSLE ^86^. The demonstration of the uptake of specific metabolites might in the future be closely associated with neuronal injury in NPSLE.

Perfusion imaging such as done with single-photon emission computed tomography (SPECT), positron emission tomography (PET) and MRI could reveal abnormalities in SLE patients. There are three techniques of perfusion MRI, based on the administration of gadolinium (dynamic susceptibility contrast imaging and dynamic contrast enhanced imaging), or without contrast administration (arterial spin labeled imaging). The main parameters derived from them are mean transit time (MTT), time to peak (TTP), cerebral blood flow (CBF) and cerebral blood volume (CBV). The defined pathological patterns are hypoperfusion (high MTT/TTP, low CBF/CBV) and hyperperfusion (low TTP/MTT, high CBV/CBF) ^87^. Few prospective studies analyzed brain perfusion in SLE patients. Some authors showed that perfusion in SLE patients was not different from healthy controls ^88^, while others reported a pattern of hypoperfusion in both SLE and NPSLE ^63^, or even hyperperfusion in the posterior cingulate gyrus in patients with active disease ^89^.

Overall, advanced MRI techniques seem to be able to detect microstructural brain damage in a very early stage when not visible on conventional sequences. There could be a temporal dissociation between the detection of damage with these sequences and its translation to significant abnormalities on conventional MRI. Advanced MRI is also expected to help to better understand the underlying pathological substrate of cerebral damage in NPSLE. However, the role of advanced MRI techniques in patients with SLE is yet in its infancy and needs to be further investigated. Future longitudinal studies should determine whether early changes of the white and gray matter in NPSLE patients may involve a higher degree of tissue-specific brain atrophy over time and to what extent it would be possible to monitor disease progression and response to therapy.

Looking at all sides of the argument, it is questionable what patients and when should be referred for brain MRI and what is the role of MRI in the clinical management of NPSLE. Syndromes such as cerebrovascular disease, cognitive dysfunction, seizures and myelopathy, as well as the focal symptomatology, were often related with radiological abnormalities, and require to be comprehensively studied; additional sequences such as DWI, GRE/SWI and contrast-enhanced should be included when MRI is performed in these patients. Conversely, MRI is more likely to be unremarkable in some other syndromes such as headache, psychosis and, generally, diffuse neurological presentations rather than focal ones. Additionally, the status of antiphospholipid antibodies, complement and disease activity plays an important role. Therefore, in the current settings, the decision of when imaging a patient remains probably best reached through a case-based clinical judgment.

The other side of the argument of MRI in NPSLE regards ruling out other causes of neuropsychiatric manifestations rather than diagnosing NPSLE. Despite MRI being the imaging modality of choice and despite significant recent advances in this field, there are neither diagnostic nor specific radiological findings for NPSLE, meaning that MRI cannot confirm nor exclude the diagnosis of NPSLE. Nevertheless, in the absence of alternative diagnoses when imaging a SLE patient, some patterns may be proposed: stroke in young patients, more than one infarct, association between large and small vessel disease, high lesion burden at young age and premature cortical atrophy. All these in a subject meeting criteria for SLE and without other risk factors, probably could suggest either the presence or a possible development of NPSLE in the following period.

In conclusion, MRI is crucial for both supporting the diagnosis of NPSLE and for ruling out alternative diagnoses. A complex diagnostic algorithm including neurophysiologic studies, laboratory tests and MRI is probably the best clinical approach. The multimodal MRI approach including conventional and advanced techniques may be an important tool for monitoring the disease activity, progression and treatment response, and may provide fundamental insights into the pathological substrate. To make this possible, a common radiological terminology is a first requirement.
