# Supplementary material for: Advanced and Conventional Magnetic Resonance Imaging in Neuropsychiatric Lupus
Source: F1000Res. 2015 Jul 28;4:162. Originally published 2015 Jun 23. [Version 2] doi: 10.12688/f1000research.6522.2 (PMC4505788; doi:10.12688/f1000research.6522.2)
Supplement: Peer review history

# Peer review: Referee response for version 2

**DOI:** 10.5256/f1000research.7385.r9693

**Published:** 2015-07-30

**Authors:** Gian Domenico Sebastiani

**Affiliations:** 1 Unità Operativa Complessa Reumatología, Azienda Ospedaliera San Camillo-Forlanini, Rome, Italy

Authors have made the suggested modifications and the paper is now suitable for indexation.

I have read this submission. I believe that I have an appropriate level of expertise to confirm that it is of an acceptable scientific standard.

# Peer review: Referee response for version 2

**DOI:** 10.5256/f1000research.7385.r9696

**Published:** 2015-07-29

**Authors:** Murray B. Urowitz

**Affiliations:** 1 Lupus Clinic, Centre for Prognosis Studies in the Rheumatic Diseases, Toronto Western Hospital, University of Toronto, Toronto, ON, Canada

I have read this submission. I believe that I have an appropriate level of expertise to confirm that it is of an acceptable scientific standard.

# Peer review: Referee response for version 1

**DOI:** 10.5256/f1000research.7001.r9164

**Published:** 2015-07-17

**Authors:** Gian Domenico Sebastiani

**Affiliations:** 1 Unità Operativa Complessa Reumatología, Azienda Ospedaliera San Camillo-Forlanini, Rome, Italy

This review is well written and give us a comprehensive updating on magnetic resonance imaging in NPSLE. 

Only few minor suggestions:

1. Introduction - Authors say that "reliable methods for diagnosing NPSLE are also unknown...". This is essentially true, but they could quote the recent contribution by Bortoluzzi *et al*. (2015), dealing with the development of a new algorithm for attribution of NP events in SLE.
2. Conventional MRI, page 2 - "DWI sequences". Please specify the meaning of DWI.
3. Conventional MRI, page 3 - "total corticosteroid". Cumulative is better.

I have read this submission. I believe that I have an appropriate level of expertise to confirm that it is of an acceptable scientific standard.

# Peer review: Referee response for version 1

**DOI:** 10.5256/f1000research.7001.r9530

**Published:** 2015-07-16

**Authors:** Murray B. Urowitz, Zahi Touma

**Affiliations:** 1 Lupus Clinic, Centre for Prognosis Studies in the Rheumatic Diseases, Toronto Western Hospital, University of Toronto, Toronto, ON, Canada

Sarbu *et al.* review the utility of MRI imaging in neuropsychiatric lupus (NPL) both what is available today in routine care and for research purposes and what might become useful in clinical care in the future. In NPL clinicians are interested in three major questions. First, is there evidence of NPL at any time in the past; secondly, is there evidence of active NPL currently; and thirdly can you demonstrate change over time with or without treatment in NPL.

In terms of the first question Sarbu *et al. *review the conventional MRI findings in the cortical, subcortical and spinal cord regions. However the nature of the morphologic changes is non specific and many could be compatible with hypertension, amyloid, Alzheimers etc. and thus may not be helpful to the clinician. Even the advanced techniques (not universally available) measuring brain volume and composition are not specific for SLE.

In terms of the second question Sarbu *et al. *review Magnetic Resonance Spectroscopy (MRS) which allows the analysis of brain metabolites. The demonstration of the uptake of specific metabolites might in the future be associated with neuron excitation but no studies are available in NPL.

Perfusion imaging such as also done with SPECT and PET scanning can reveal areas of hyper or hypoperfusion but thus far no studies demonstrating active NPL which could change over time exists.

In conclusion, this article is a good review of what currently exists and what may be developed over time with MRI imaging. The authors conclude that further clinical association studies with conventional and advanced techniques are required before MRI imaging will become the standard to understand the mechanism, diagnosis and monitoring of NPL.

We have read this submission. We believe that we have an appropriate level of expertise to confirm that it is of an acceptable scientific standard.

# Peer review: Referee response for version 1

**DOI:** 10.5256/f1000research.7001.r9358

**Published:** 2015-07-07

**Authors:** Fabiola Atzeni

**Affiliations:** 1 Rheumatology Unit, Luigi Sacco University Hospital, Milan, Italy

**Title and Abstract**: The title is quite generic and perhaps the description of old and new techniques should be emphasized.

**Article content**: Design, methods and analysis are well explained and appropriate, however the use of nanoparticles (e.g. SPIONS) should be mentioned among the new MRI techniques.   

**Conclusions**:  The conclusions underline the need for MRI imaging in symptomatic neuropsychiatric SLE, although a standardized consensus still lacks. It should be more evidenced, however, the utility of a complex diagnostic algorithm, including neurophysiologic study of the brain or lab tests, beyond the solely use of MRI.

**Data**: Methods and indications to a specific procedure are well described ; MRI findings are reported according to pathologic or clinical pictures.

I have read this submission. I believe that I have an appropriate level of expertise to confirm that it is of an acceptable scientific standard.
